# Understanding the presence of military priests conducting military soul care in the Swedish armed forces: a medical sociological perspective

**DOI:** 10.3389/fsoc.2024.1408067

**Published:** 2024-11-29

**Authors:** Jan Grimell

**Affiliations:** Department of Sociology, Faculty of Social Sciences, Umeå University, Umeå, Sweden

**Keywords:** military chaplaincy, military spiritual care, military soul care, military pastoral care, Church of Sweden, militär själavård, fältpräst

## Abstract

In the 20th century, military medicine and psychiatry emerged as dominant paradigms in Western military contexts, shaping practices across recruitment, selection, training, screening, evaluation, diagnosis, and treatment. This approach paralleled trends in broader Western society, where it has faced criticism for medicalizing social, psychological, spiritual, and existential issues, often applying methods ill-suited to address certain forms of suffering. Despite this shift, alternative approaches embodied by military priests have maintained a meaningful role in the Swedish armed forces, in a country often, if somewhat misleadingly, regarded as one of the world’s most secularized. This article aims to elucidate the foundations, concepts, distinctiveness, and contributions of military soul care—*militär själavård* in Swedish—as practiced by military priests, or *fältpräster*, within the Swedish military context. Unlike chaplains (ordained and others) in other Swedish settings (e.g., hospitals, prisons, schools, airports, police) or military chaplains internationally, Swedish military priests operate within a unique mandate and purpose. Military soul care aims to build resilience to crises and wartime conditions, strengthening individuals’ will to defend and fight. The framework encompasses military soul care and counseling, advisory roles, education, ceremonies (including field sermons, prayers, memorials), and support for commanders in times of crisis. While military medicine and psychiatry continue to hold normative authority in a Swedish military society, there is a growing recognition that military personnel require more than physical conditioning to build mental resilience. Enduring the complex stresses of warfare demands approaches that transcend medical perspectives, addressing the human condition within a cultural and symbolic context. This article highlights key historical, societal, military, and ecclesiastical perspectives essential for understanding why this distinctive approach to military soul care has emerged in Sweden.

## Introduction

The power dynamics of modern medicine have arisen in a complex social process which involves various forms of *surveillance* (Foucault, 1979, 2006), such as classification, exclusion, individualization, normalization, and totalization (Gore, 1995); deviance and social control (Parsons, 1991); and commercial interests (Bradby, 2012). This process, which involves an interaction between society, medicine (as profession, discipline, business, statutory, and non-governmental institution), and individual needs for (medical) attention, has led to a growing tendency to *medicalize*, or to diagnose and medicate suffering (Conrad, 2005; Conrad et al., 2010) to such an extent that medical intervention may harm through error, side-effects, addiction, and drug interactions—a phenomenon described by Illich (1977) as *iatrogenesis*. Thus, the tendency to over-medicalize social, psychological, spiritual, and existential problems suggests that medicine treats forms of suffering for which its methods are ineffective (Bradby, 2016). *Surveillance* (Foucault, 1979)*, medicalization* (Conrad, 2005; Conrad et al., 2010), commercial interests (Bradby, 2012), and human needs for (medical) attention (Bradby, 2016) are helpful perspectives to unfold and understand a social process which has given birth to the power position that medicine has in today’s Western society, as well as its undesired side-effects; *iatrogenesis* (Illich, 1977). By seeing health and illness as social as well as individual bodily processes, and conceptualizing medicine as a practice and profession that is entangled with governance and speculative capital, sociology offers critical insights to medicine’s curative and therapeutic benefits (Bradby, 2012).

### Modern medicine and psychiatry

The two world wars in the 20th century had great significance for the advancement of modern medicine (Bradby, 2016) including the dominant role of psychiatry in classifying and understanding the healthy and sick (Foucault, 1979, 2006)—especially in the military context, something that also came to exert influence in the psychiatric arena outside of the military (Moss and Prince, 2014). The development within military psychiatry had a particularly expansive curve during these wars and then continued to consolidate its position within armed forces around the world, a development which has been carefully presented in a post-structuralist approach by Moss and Prince (2014). Two major processes came into play here: the military’s strong need for functioning military personnel in war, and psychiatry’s emerging claims to research and define what was perceived as “healthy” or as “sick.” Today, military psychiatry is the leading and defining knowledge paradigm and practice in a Western military context, spanning from recruitment, selection, and training to the screening, evaluation, diagnosis and treatment of illness (Moss and Prince, 2014). Yet, despite this development, there is still the need for a contrasting cultural element in military organizations that base wisdom, experience, and practice on a completely different ontology and epistemology—namely military spiritual care and military chaplains (ordained and otherwise).

Military psychiatry today embodies both power, as described by Foucault (1979, 2006), and the accepted standards of what it means to be *good* and *healthy* versus *unhealthy* and *sick* within Western military organizations and among veterans (Kilshaw, 2011; Moss and Prince, 2014). Despite this, military chaplains continue to play an important role in addressing the existential and spiritual complexities of war. This role is significant within military organizations and among veterans across the Western world (Graham, 2017; Grimell, 2020a; Grimell, 2020b; Koenig et al., 2023; Lee, 2018; Liuski and Grimell, 2022; Smith-MacDonald et al., 2018; Stallinga, 2013; Wortmann et al., 2017). The ontological and epistemological perspectives on life, humanity, existence, health, and suffering, embodied and communicated by military chaplains, are not considered outdated. This is particularly true in the context of conflict and war, where these perspectives remain deeply relevant (Stallinga, 2013).

While military psychiatry today embodies both power (Foucault, 1979, 2006) and the accepted truth about what it means to be good and healthy and what is unhealthy and sick in Western military organizations and among veterans (Kilshaw, 2011; Moss and Prince, 2014), military chaplains continue to play an important role, given the existential and spiritual complexities of war, in military organizations, and among veterans around the Western world (Graham, 2017; Grimell, 2020a; Grimell, 2020b; Koenig et al., 2023; Lee, 2018; Liuski and Grimell, 2022; Smith-MacDonald et al., 2018; Stallinga, 2013; Wortmann et al., 2017). Accordingly, the ontological and epistemological views of life, humanity, existence, health, and suffering, embodied through and communicated by military chaplains, are not seen as obsolete and relegated to a time when we did not know any better—especially not in the context of conflict and war (Stallinga, 2013).

### Military spiritual care and chaplaincy services

This interesting relationship between military medicine and psychiatry on the one hand and military spiritual care and chaplaincy services on the other constitutes an important field of investigation for a progressive sociology of medicine which aims to critically approach the range of interests that make up medicine while holding a sense of medicine’s benefits and deficits at individual and system levels in balance with other knowledge systems and moralities of care, support, and healing (Bradby, 2016).

Today there seems to be a growing curiosity within especially military psychiatry and psychology for military spiritual care (see, for instance, Bobrow et al., 2013; Bryan et al., 2015; Koenig et al., 2023; Tick, 2005; Wortmann et al., 2017). This has several layers, where the intensified research interest in the multifold challenges for deployed military personnel and veterans during the war on terror including, in particular, moral conflict and injury (Atuel et al., 2021; Shay, 2002; Litz et al., 2009), has re-actualized military spiritual care (Wortmann et al., 2017).

However, approaching and dialoging with military spiritual care from a psychiatric and psychological perspective holds several challenges in terms of ontology, epistemology, methods and methodology, and even contextuality. While there are benefits (Smith-MacDonald et al., 2018) there are also risks (Moss and Prince, 2014) to consider when medicine (clinicians) and military spiritual care (ordained chaplains and others) dialog and cooperate. Because power dynamics are still operational, this risks being reflected in, for example, a desire to classify and understand military spiritual care from a medical lens—not on its own ontological terms—or undermining the integrity of the absolute secrecy.

### Aim

This article aims to clarify and present the foundations, concepts, uniqueness, and contributions of military soul care in a Swedish context through a medical sociological lens. Military soul care could be defined as an ecclesiastical-military practice conducted solely by military priests (*fältpräster* in Swedish), aimed at strengthening the will to fight among military personnel.

### The framework of military soul care

The framework of *military soul care* is understood as a distinct concept, separate from the *soul care* practiced within the Church of Sweden (which, in the Swedish context, can be translated into English as pastoral and spiritual care and counseling). This distinction pertains to, but is not limited to, historical, and ecclesiastical aspects, as well as the purpose of this practice within the Swedish Armed Forces.

Sweden is a unique context, for several reasons, when it comes to military soul care. Priests from the Church of Sweden have been a salient and active element in the Armed Forces in an unbroken line since the country was founded as a modern national state in the 16th century. This likely makes Swedish military chaplaincy services the oldest institution of its kind within any national armed forces in the Western world. Research shows that Swedish military chaplains have traditionally held a prominent role in the armed forces (Grimell, 2020a). They conduct military soul care with a more comprehensive mandate than that of other nations (Grimell, 2020b; Grimell, 2021b). Furthermore, the institution is expanding significantly, growing from an already large organization with around 100 military priests to an even larger one with hundreds more, in line with the extensive expansion of the Swedish Armed Forces (Grimell, 2023a). This power dynamic contrasts with other chaplaincy services in Sweden (e.g., in hospitals, prisons, airports, juridical settings, schools) and military chaplaincy services elsewhere^1^.

The article will begin by exploring the emergence of military soul care, starting with a historical perspective on the topic. It will then examine the power position of the former state church and its relationship with the armed forces, followed by an analysis of the concept of military soul care both internationally and nationally, in relation to other concepts and military medicine. Finally, the article will reflect on how ecclesial and military cultures intersect for priests through a hybrid identity.

## The emerging identity of Swedish military soul care: a historical perspective

During the 16th century, Sweden became a united and independent kingdom with a royal power that was inherited and a church that obeyed the king. Gustav Vasa was the unifying force, and under his sons Sweden’s importance in Europe increased, even though Gustav’s sons fought each other for the crown. Gustav’s role in introducing a Swedish hereditary monarchy is seen today as the founding of Sweden as a modern nation-state (Larsson, 2002; Ringmar, 2002). Sweden’s national day is June 6, when Gustav was elected king by the Riksdag (Parliament) in Strängnäs in 1523. When Gustav organized the nation’s armed forces during the 16th century, the church’s Lutheran priests also became part of forces on land and at sea. This marks the start of what we today call “military chaplaincy services,” and priests from the Church of Sweden been serving in an unbroken line in the Swedish Armed Forces ever since (Grimell, 2020a; Grimell, 2020b).

### The role of field sermons and field prayers during the 17th and 18th centuries

Historical research^2^ (Gudmundsson, 2014) has investigated the themes and functions of field sermons and field prayers in the Swedish army between 1611 and 1721. At this time Christianity was imperative in the Swedish context and the armed forces were explicitly confessional. Gudmundsson (2014) has suggested that the basic functions of field sermons were 2-fold: discipline and comfort. The disciplinary aspects were understood in terms of the fundamental Lutheran view of the social order, which was expressed in the doctrines of the three estates and vocation. The army needed disciplined soldiers, and field sermons responded to this need; not primarily in response to the needs of the army, but as a reflection of Lutheranism’s worldview and self-understanding. The main expression of the sermons’ comforting function was the Gospel of Christ. This meant that the soldiers should not fear death, because if they were to die, it would be the will of God, and after death awaited eternal life. Preaching helped to shape the self-image and worldview of both officers and soldiers in the Swedish Army in three ways. A confessional Lutheran identification as the right and correct sort of Christian was set against *the confessional others* (at that time—shortly after the Reformation in Europe—confessional others were illustrated by the conflict between Lutheran and Catholic countries and allies). An important part of a national identification as a Swedish subject was the belief that they were the chosen people. A professional identification as a Christian soldier was based on the Lutheran doctrines of vocation and of the three estates, which meant it was legitimate for a Christian to be a soldier. The soldierly ideal was fundamentally a Christian ideal. To sum up, field sermons and field prayers served functions of discipline, comfort, and positive self-image which in turn, whether directly or indirectly, fueled soldiers’ will to fight and their ability to stay committed to combat and the cause of the war.

### Military chaplaincy during the 20th century

More recent historical research (Hansson, 2016) about military chaplaincy services care during 20th century testifies to a continuation of the function of the early era. Hansson (2016) describes through documents and written sources the organizational development of military soul care during the 20th century. In particular, Hansson (2016) highlights the purpose of military soul care in two principles: the principle of effectiveness, the purpose of which was that the soul care should benefit the effect of the unit; and the principle of representation, which aimed to meet individual religious, ethical, and cultural needs as far as possible. While Hansson (2016) speculated that the principle of effectiveness may have expired during the early 2000s, recent empirical research suggests otherwise (Grimell, 2020b; Grimell, 2021b). In fact, the official position of the Swedish Armed Forces’ Chief of Chaplains and Brigadier General Ahlén^3^ and the Swedish Armed Forces (2024) is that the function of military soul care is to nurture resilience through spiritual, existential, moral, and ethical means, and through military church traditions like field sermons so as to support soldiers’ ability and will to fight.

### A common thread for military soul care during the 16, 17, and 20th centuries

Against the background of this historical perspective and reasoning, it is possible to detect a common thread spanning from the birth of the military soul care in the 16th century to today in terms of what it ultimately aims for: spiritual comfort, moral resilience, and battlefield effectiveness by enhancing the will to fight through cementing an existential and cultural perspective. In this regard, we can talk about a special kind of Swedish military soul care identity which emerged when the nation was founded, and military priests were called by the king to support and contribute to the armed forces. This identity of the Swedish chaplaincy services differs significantly from many other countries, where military spiritual care is mainly centered on the individual’s right to practice his or her religion in the armed forces and where the armed forces are obligated to meet these individual needs through diverse and pluralized military chaplaincy services. In contrast, the Swedish military chaplaincy service is rooted in a different tradition, is supported by other assumptions and purposes, and is staffed exclusively by military priests from the Church of Sweden.

The Church of Sweden has thus been embedded in the Armed Forces for almost 500 years, and research shows that the soul care activities, whether they occur in the field, in prayer rooms, in churches, or during deployments, can be described as a kind of *lived religion* (McGuire, 2008) with deep roots in the military contextual tradition and the former state church (Grimell, 2020a; Grimell, 2020b; Grimell, 2021b).

### A military culture with a certain resilience and resistance in relation to broader society

An interesting observation is that, in the late modern period, the military context and culture have proven particularly resistant to the secularization and pluralization that have occurred in other governmental agencies and societal contexts. The archaic feature of military culture—i.e., to train to kill and to kill other people (combatants) on behalf of the state—combined with a resistance to change (i.e., military cultural conservatism) has molded a Teflon-like cultural context which operates on its own terms (Grimell, 2021a, 2022, 2023b). This can be contrasted with Swedish healthcare chaplains, who operate under very different conditions (Grimell and Bradby, 2022). The healthcare context is highly pluralized and secularized, with medicine as the defining cultural standard. Healthcare chaplains must work constantly to be seen and make their presence known in a busy and stressful healthcare context that puts all the focus on medical care. Additionally, the absolute confidentiality requirement is not as pronounced in a Swedish healthcare context, as there are chaplains from a variety of faiths and spiritual traditions, each with different approaches to this concept. At the same time, for example, there are deacons who serve as healthcare chaplains and have a somewhat more lenient approach to confidentiality (see the next section where this is further explored). Thus, the role and function of healthcare chaplains is in contrast to military priests (Grimell and Bradby, 2022).

## The Church of Sweden, its priests, and the armed forces

Until 2000, the Church of Sweden was a state church, but has since been divorced from the state. However, even though 24 years has passed since the separation between the church and state, the Church of Sweden is to the highest degree still politically governed and controlled at national, regional, and local levels. The way the church is organized between administrative staff in the broader sense and clergy, including in decision-making processes, appears to be based on the same principle of division as political organizations which operate at national, regional and local levels. All major decisions are made by politically elected representatives, which means that the Church of Sweden largely reflects society’s political representations and values. Even the threefold ministry (bishop, priest, deacon) is shaped by political decisions in the parliamentary *Kyrkomötet* (the annual Church Meeting) on the national level (see the Order of the Church, 2024). Social Democrats, as is the case in Swedish society, constitute the largest political organization in the Church of Sweden (Kyrkans Tidning, 2021). Although Sweden—in a methodologically questionable and sometimes blunt way—is often described as one of the world’s most secularized countries (Lemos and Puga-Gonzalez, 2021; also see World Value Survey Cultural Map, 2024), there are still 5.5 million members in the church; i.e., more than half the population (Church of Sweden in Statistics, 2024).^4^ Members of the Church of Sweden do not attend services regularly, though they often participate during traditional highlights such as the First Sunday of Advent and Christmas. They particularly value the church’s social work. Many have grown up in a broadly Swedish ecclesiastical culture where the church has historically held, and continues to hold, an important place and voice in society. Membership is based on individual choice, with each member determining their own level of participation.

### A church with a ministry-like hierarchical structure and organization

Additionally, the Church of Sweden at the national level still manifests a ministry-like superstructure—a ministry-like Church Office with over 500 employees and 11 departments, plus approximately 100 employees abroad (National Church Office, 2024). This Church Office is bigger than many governmental ministries in Sweden, even bigger than the Ministry of Finance (Government Offices in Sweden, 2024). The only thing missing is a politically elected minister to report to and obey. Thus, the Church of Sweden continues to function, to a large extent, as a state church and has a special status and position in the country, even if the state and the church are separated in a strictly formal sense.

Military chaplains in the Swedish Armed Forces have been priests from the Church of Sweden since the 16th century (Gudmundsson, 2014). Apart from what has already been presented about the impact of history, political design, and structure in understanding military chaplaincy services and military soul care in Sweden, there are additional reasons for this. Each reason constitutes an important part of the whole and the following aspects need to be considered in the Swedish case.

### The traditional and cultural perspective

First, is the cultural perspective; the relationship between the church and the armed forces is based on an almost 500-year-long unbroken tradition of priests from the Church of Sweden (Gudmundsson, 2014; Hansson, 2016). These priests do not only represent the church but represent and maintain deeper aspects of societal vales, meanings, and practices—i.e., culture itself, a culture which can be said to have been shaped and reshaped since the nation of Sweden was created. The church and its priests are representatives and intermediaries of Swedish church rites, traditions, and values that are deeply embedded in society at large and particularly in a Swedish military cultural context (Grimell, 2021a). So, priests do not only convey the explicitly ecclesiastical aspects of soul care but also handle and communicate culture and a collective memory rooted in a long Swedish tradition to such an extent that their presence and representation should also be understood from a cultural and more general Swedish tradition-bound identity perspective. This is rarely or never articulated in international research on military spiritual care, which is largely due to the fact that the historical context and its impact is so different between countries, and Sweden stands out as an outlier in this regard. This is also why sweeping generalizations about military spiritual care may be too broad and risk missing the contextual and unique cultural nature of any one country’s military spiritual care and chaplaincy services.

### The perspective of the (former) state church

Second, is the (former) state church perspective. The Church of Sweden is organized according to the territorial congregational principle, which means that every geographical area in the whole of Sweden is divided into congregations and each congregation has a pastoral responsibility for everyone who stays in this area, no matter the duration of their stay, including military personnel and military units (Order of the Church, 2024, Second Division, Congregation, Chapter 2, Paragraph 1). This means that according to the territorial congregational principle and the concept of presence/residence, the Church of Sweden has employed priests who in their ministry have pastoral responsibility for military personnel and units. Beyond this principle, which provides for military soul care, the armed forces can also employ priests to serve as military chaplains in the Home Guard (often outside regular working hours on weekends), during deployments to conflict zones (for a limited number of months), and within the military organization designed to operate during wartime. During deployments, priests apply for a temporary leave from their ecclesiastical employer for the period of their deployment (for a full account of various Swedish military chaplaincy positions see Grimell, 2020a; Grimell, 2020b). Although the Swedish Armed Forces have a Chief of Chaplains (financed by both the Armed Forces and the Church of Sweden), it is the respective bishop of the diocese in which the priest serves who has ecclesiastical supervisory responsibility and who, according to the Order of the Church (2024), can even declare a priest ineligible to exercise the office if s/he violates their ordination or vows (Order of the Church, 2024, Thirteen Division, Supervision and appeal). The Church of Sweden owns the threefold ministry (of deacon, priest, and bishop) and has absolute power—via the ecclesiastical judicial authority on the regional level for priests and deacons, and for bishops on the national level—when it comes to who has the right to exercise the ministry of a priest (a deacon and a bishop). But the Chief of Chaplains forms the mediating link between the Archbishop of the Church of Sweden and the Supreme Commander in the Armed Forces. At regional level, so-called region military priests support and advice the dioceses and military regions, and on the local level, military priests support and advise regiments, flotillas, and bases, as well as parishes and congregations. The relationship between the armed forces and the Church of Sweden can rather be characterized as a kind of collaboration or cooperation between two of the “big” societal institutions.

### The perspective on the requirements and qualifications to serve as a military chaplain

The third perspective refers to requirements and qualifications. While the official approach of military chaplaincy in Sweden is to be ecumenically open, the requirements of military chaplains are so high that in practice no one besides priests from the Church of Sweden serve as chaplains in regiments, flotillas, bases, and occupations. To exemplify these requirements, the military chaplains need to be employed as a priest, pastor, or minister by a congregation or parish and within his/her ministry be allowed to exercise part of the ministry as a military chaplain (the other part within the congregation or parish). In practice, free churches cannot afford this and do not have any particular interest in their pastors serving in a military context. The formal requirement to be accepted as a military chaplains involve a four-year theological master’s degree, plus a year of pastoral training and additional years of work experience in a traditional congregation or parish. Few pastors have these formalities in addition to the above requirements. In addition, military chaplains must also share and support the values that the Swedish Armed Forces, as a governmental agency, stand behind; for instance, LGBT rights. A different theological position does not trump the Armed Forces’ values in this case. Once again, military soul care in Sweden is not about the individual’s right to exercise their religious rights, but has a broader mandate and different purpose. Finally, there is absolute secrecy or confidentiality, which is regulated as a concept by the Order of the Church (2024), Seventh Division, Ecclesiastical Assignments and Positions (Chapter 31, Paragraph 9). There are no alternative interpretations of absolute secrecy or confidentiality in the armed forces—it is synonymous with the Church of Sweden’s definition. This means that, for example, deacons in the Church of Sweden find it difficult in practice to be military chaplains because they have a softer formulated confidentiality in the Order of the Church (2024) (Seventh Division, Ecclesiastical Assignments and Positions, Chapter 32, Paragraph 9) than priests, in combination with the fact that they cannot administer sacraments such as Holy Communion and conduct church ceremonies such as (church and field) funerals, confessions, absolution/forgiveness of sins, the blessing, etc. Furthermore, a priest can never be released from anything said under absolute secrecy, even if the person who disclosed it wishes to release the priest from the obligation of absolute confidentiality.

### The perspective on military-specific context and requirements

The fourth perspective regards the specific military context and security. There is a security aspect to consider in the armed forces, where military chaplains need to meet the requirements and security classification that may be relevant for various positions in a military organization. There are also military documents which state that the provider of military priests to the armed forces is the Church of Sweden.^5^ Again, and importantly, military soul care does not refer to the individual’s right to practice an individual religion and thus suggest that the Swedish Armed Forces must cater to this. Military soul care is based on different premises and has a different purpose, such as building resilience to crises and wartime conditions, and strengthening individuals’ will to defend and fight.

## Military soul care dialog with the spiritual, existential, and pastoral

The inclusive concept of military spiritual care—i.e., to spiritually care, counsel, and support service members, veterans, and units through military chaplains (ordained or otherwise)—is an internationally well-recognized concept. Not least because the concept of military spiritual care and support is open enough to include a wide range of spiritual perspectives and caregivers. Thus, spiritual care can be tailored to very open and loose definitions of spirituality such as the search for meaning and significance in life (Pargament, 2008, 2011), and resonates very well with concepts such as military spiritual fitness (Pargament and Sweeney, 2011) or somewhat more practical theologically opened definitions which connect spirituality to a divine or transcendent dimension (Ganzevoort, 2009; Ganzevoort and Roeland, 2014; Graham, 2017). Given a Swedish context, many Swedes can be described with the analytical concept of *cultural Christians* (Grimell, 2023a; Kasselstrand, 2015). The Swedish concept of a cultural Christian includes citizens who grew up in a time where the Church of Sweden was a state church and in whose rites and activities a large part of citizens participated during their childhoods, adolescence, and later in life (Kasselstrand, 2015). This concept applies to a kind of wider understanding of spirituality and existential curiosity and a certain cultural connection to the Church of Sweden. The generic concept of military spiritual care resonates well with such an existential and culturally ecclesiastical Swedish contour.

However, in the Swedish context, military pastoral care also proves to be a useful concept to describe the method and activities of the chaplaincy, because a priest as a military chaplain operates by the Order of the Church (2024) and exercises a pastoral responsibility over all military personnel present on a regiment, flotilla, or base, which is especially manifest during deployments. All present service members in the unit illustrate the flock which the military chaplain must then pastor (Grimell, 2020a; Grimell, 2020b).

Military priests in a Swedish context use a broad toolbox in their practices. This toolbox ranges from existential conversations about life and questions regarding existence to providing care and support through specific military church traditions, such as field sermons (Grimell, 2020b). Thus, the broader existential approach to support, care, and counseling can align with a military spiritual care perspective. At the same time, this practice is also highly influenced by regulations in the Order of the Church (2024), the identity of being an ordained priest in the Church of Sweden, and the expectation that priests in the military will pastor a military flock (Grimell, 2021a).

Additionally, in Sweden, traditional (non-military) spiritual and pastoral care operates with contrasting mandates and purposes, as these practices do not aim to increase the will to fight in any way. In this intention, there can be tension, more or less articulated, between the ecclesiastical ministry declared by the vows made during one’s ordination as a priest in the Church of Sweden and the military aim and purpose of military chaplaincy (Grimell, 2020b, 2021a). This will be discussed in more detail later; but first, let us explore the concept of soul care.

## Reasons why it has come to be called soul care in a Swedish context

The official term and the correct concept for what priests and deacons as chaplains do is *soul care* or *själavård* in Swedish (Order of the Church, 2024, Seventh Division, Ecclesiastical Assignments and Positions). The focus here is on the concept of the soul, which is a frequently recurring theme in the Swedish Evangelical-Lutheran tradition and the Order of the Church (2024). However, it is not a well-developed or precise concept. This is, in part, the point because neither the soul nor soul care can be properly defined (Kurkiala, 2019). The soul is unique to every person, a bodiless reality (Foucault, 1979). The soul transcends a human and connects us to something greater than life. The concept of the soul is a generally accepted phenomenon in a Christian tradition as well as in secular research (see, for example, Braidotti, 2006; Foucault, 1979; Moss and Prince, 2014; McLaren, 2002; Tick, 2005). So, instead of the concepts of spiritual and pastoral care, soul care is the central and vital concept in the Church of Sweden and the Order of the Church (2024). Caring for and counseling a human’s soul is what priests and deacons, as chaplains, *do* in Sweden. When these notions of soul and care are integrated, we are talking about the very core of the tradition in which clergy engage and provide support, care, and counseling/interpretation within the Swedish framework called *soul care* (Order of the Church, 2024, Seventh Division, Ecclesiastical Assignments and Positions).

### The lack of movement toward psychologization (clinical pastoral education)

It is important to stress that in the Swedish context, there has been no movement toward the psychologization of soul care which took place in the middle of the 20th century in a North American context, and which came to exert a strong influence on conceptual development and pastoral psychological and clinical pastoral education (CPE) and requirements. Capps and Fowler (2010) have written about the psychologization of pastoral theology and pastoral psychology in a North American context. Capps and Fowler (2010) traced the change of terminology and concepts to a shift tailored to the modernization of the healthcare sector in USA. When pastors were given a place and space in the healthcare sector during the modernization of the healthcare sector beginning around 1940, concepts and terminology also came to be adapted and changed, given the overall normative secular context. Classical expressions such as the *cure of souls* (Boisen, 1936) were neutralized and took on more of the language of secular psychology. Instead of the *cure of souls*, the tone-setting pioneers in pastoral psychology Cabot and Dicks (1936) and Dicks (1944) coined the concepts *pastoral work* and *pastoral counseling*, concepts that remain dominant today. The CPE movement was brought to Europe via the Netherlands and Germany, where it gained influence, just as in Norway and Finland, becoming a formalized and recognized part of the church’s spiritual work (Saarelainen et al., 2019; Thomsen et al., 2019). The influence of CPE, however, never made it to either Sweden nor Denmark partly because it seemed foreign to Swedish and Danish theological education and attitudes after World War II, which was orientated to the influential theological thinkers of their respective countries around this time who also addressed topics such as pastoral care in their theological approaches (Thomsen et al., 2019; also see Danbolt, 2019, Special Issue on Chaplaincy in a Northern Europe).

The powerful influence of behavioral science illustrates a certain power dynamic which has, both consciously and unconsciously, moved terminology away from the cure or care of the souls, particularly in secular settings, and as a result adapted the terms and concepts of psychology and psychiatry. Is such an adaptive process, governed by the power dynamics of medicine (psychiatry and psychology included), genuine concepts and expressions may have been lost to varying extents, also influencing the more clinically oriented approaches to pastoral care and counseling (Capps and Fowler, 2010; Capps, 2008; Thomsen et al., 2019).

### The maintenance of a deeply ingrained concept, tradition, and practice

In Sweden, on the contrary, the choice to keep traditional and ingrained concepts has remained firm. Thus, soul care, a deeply ingrained concept in the minds and folk knowledge of Swedish citizens and agencies, has resisted the power dynamics of medicine. Accordingly, what Swedish military priests do, formally and technically, is *military soul care,* and the same applies to all priests and deacons and chaplains (from the Church of Sweden) regardless of whether they are in a hospital, prison, school, airport, congregation, or elsewhere. What priests, in a strictly conceptual terminology practice—the method or approach—is soul care and this is defined and regulated in Order of the Church (2024) and is the concept used in the official (secular) website, published, for instance, by the Swedish Armed Forces (2024). In addition, the term “military chaplain” does not adequately resonate with the practice and concept of military soul care in Sweden. First, military soul care includes, by necessity, absolute secrecy which can only be upheld by a priest. Thus, the most suitable concept would be a military priest in a Swedish context, and this is also the formal term (along with other suffixes such as “Home Guard priest”) for a priest who conducts military soul care within regiments, flotillas, bases, and in the Home Guard across Sweden (Grimell, 2021a). Second, a priest is also titled a “provider” of soul care or soul care giver in the Swedish context; the one who conducts soul care to/on a care receiver or confidant. Again, this is also regulated in the Order of the Church (2024), as well as in the terminology used by secular agencies and exists in public knowledge. However, when it comes to the healthcare sector, there is currently a tendency among some secular agencies as a result of a dominant medical cultural and pluralization to adapt the language slightly. For instance, the Swedish Agency for Support to Faith Communities has moved more toward the inclusive expression *spiritual care*, in order to have a wider appeal to minor faith communities that do not use the term soul care, even if the term is explicit on their official website in Swedish (Swedish Agency for Support to Faith Communities, 2024). An agency such as this operates with a completely different mission in mind, where it is important to support individual faith communities and the rights of individuals to practice their religion; for example, during a hospital stay.

To sum up, a reasonable contextual conclusion is that as a rich, well-organized and powerful former state church with 5.5 million members, the politically governed Church of Sweden has not felt forced to adapt or subordinate itself to the power dynamics of medicine in order to have a role and place in secular contexts outside of the church setting (e.g., in a military, prison, school, hospital, etc.) On the contrary, the concept of soul care and absolute confidentiality have been an important way to maintain integrity and offer something truly unique while distancing oneself from the paradigm of *surveillance* (Foucault, 1979, 2006), *medicalization* (Conrad, 2005; Conrad et al., 2010), *iatrogenesis* (Illich, 1977), and commercial interests (Bradby, 2012), as priests and deacons direct attention to the human needs for soul care. Nor has a North American Clinical Pastoral Education (CPE) movement or training (Capps and Fowler, 2010) gained a foothold in a Swedish context, as it has elsewhere.

The framework of contemporary military soul care in the Swedish military context.

### Military soul care in the 2020s

Military soul care as a concept can be defined both narrowly as the activity dealing with care and counseling tailored to individual soul care and confession, and at the same time very broadly as an umbrella concept that organizes all types of activities that a military priest exercises in a military context. Although these activities may have a public character with specific military names such as field sermon (*korum* in Swedish) or church-specific terms in accordance with the Order of the Church (2024), they are organized within the framework of military soul care as an overall conceptual approach. According to the Church of Sweden, all activities must be imbued with a soul care approach, which also makes it possible to propose in a military context the concept of military soul care as an organized principle for all activities; i.e., everything that is done is aimed at military soul care.

On the Swedish Armed Forces (2024) official website about military soul care, it is stated that it has existed as a deep insight for a very long time that military personnel need more than just physical training in order to successfully defend the nation of Sweden and our values. The Swedish Armed Forces’ official stance is that a strong ethical value base is absolutely necessary for military personnel to be able to act and make decisions based on good ethics and morals under heavy pressure. Military soul care has the task of strengthening this and contributing to the feeling of (a cultural) sense of coherence (SOC, see Antonovsky, 1987), which creates conditions for the individual to feel meaningfulness, manageability, and comprehensibility (Swedish Armed Forces, 2024).

Moreover, it is stated that military soul care aims to increase an individual’s ability to perform, as well as their mental strength and moral resilience (Swedish Armed Forces, 2024). Military soul care serves to support spiritual balance and good existential health and strives to increase an individual’s endurance of the existential and moral stresses of crisis and war. This is important for the Swedish concept of the psychological defense as well as in strengthening the will to defend the country. Military soul care is inclusive and is carried out unconditionally regardless of faith community affiliation or faith. Military soul care supports and promotes humanity, equality, and the Swedish Armed Forces’ core values (e.g., LTGB rights) despite differences (Swedish Armed Forces, 2024).

### The five duties that constitute military soul care

**Figure 1:**
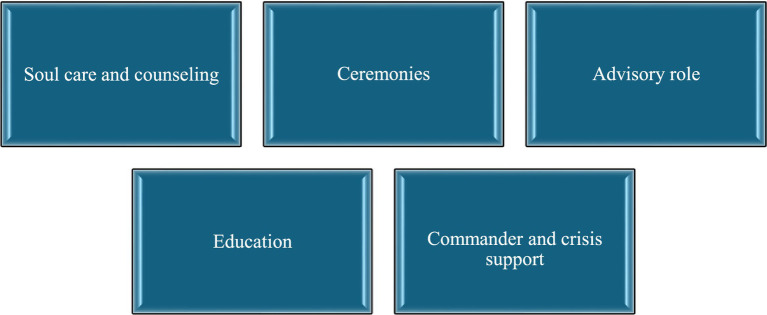
Conceptualization of the five duties performed by military priests within the framework of military soul care in the Swedish Armed Forces.

Military soul care in the Swedish Armed Forces,^6^ conceptualized in Figure 1, includes five formalized duties (tasks or missions). The duties exist without an internal hierarchy and each military priest must be ready to meet every aspect of military soul care. Some duties may be more pronounced than others depending on the context(s), situation(s), and need(s).

Soul care and counseling are tailored to ensure absolute confidentiality. Ceremonies, which include but are not limited to field sermons, field prayers, and memorials, are an integral part of this care and contribute to a sense of coherence. The advisory role encompasses providing guidance in both military contexts and within the Church of Sweden, particularly regarding matters like war preparedness. Education within this framework involves training in ethics, morality, and war grave services. Additionally, commander and crisis support plays a crucial role in assisting commanders, especially when a soldier dies in combat or in the event of a serious accident.

Additionally, the overall mission of the military priest function—at the Chief of Chaplains’ level—is to serve the armed forces during peace, crisis, and war, in the military organizations that train personnel, as well as in the wartime organizations designed to wage war.

### A unique practice that operates on its own cultural foundation

The uniqueness of military soul care in the Swedish context is that there are neither military medicine (psychiatry and psychology included) nor other faith communities which can conduct military soul care given its ontology, conceptualization, requirements, regulations, purposes, and cultural aspects. This is a phenomenon embodied by the Church of Sweden, where almost 500 years of accumulated knowledge and wisdom from the church’s tradition as a whole (not just soul care), including theological and pastoral training as well as the robust experiences of priests (in an ordinary congregations) who meet various people in all stages of life (in joy, crisis and sorrow) comes to the Swedish Armed Forces for its benefit. Military soul care also allows a fully protected place to vent for military personnel and veterans where they can talk under the protection of absolute secrecy completely freed from the surveillance paradigm (Foucault, 1979, 2006). A priest from the Church of Sweden may also not be called to testify in a trial about things that the priest has learned during confession or during individual pastoral care (Church of Sweden in Statistics, 2024; Courts of Sweden, 2024). This has been regulated in Swedish law for a long time.

Although the educational explanation of military soul care on the Swedish Armed Forces’ official website involves broad and inclusive words such as spirituality, existential health, psychological defense, and the will to defend one’s country, these should be understood as precisely educational words to describe in broad terms what military soul care includes, implies, and entails. Military soul care—and soul care in general—should be understood on its own ontological and epistemological terms; i.e., soul care should not be understood or approached from behavioral science assumptions and randomized test–control group principles. The soul cannot be captured or studied empirically; therefore, soul care should not be subjected to such an empirical approach. In other words, studying and measuring the effects of soul care, both narrowly and broadly defined, is problematic for several reasons, of which a few are outlined below:

- Military soul care, narrowly and broadly defined, represents activities that are deeply culturally embedded and cannot meaningfully be distinguished from military and societal cultural tradition because they symbolize values, meanings, and practices that tell us about who we are as the country of Sweden and what we stand for.
- Military soul care rests on ontological assumptions that are based on something that is bigger than humans and transcends our being and which cannot be epistemologically measured in a meaningful way, even if it can exert a meaningful effect on humans—i.e., reductionist social science approach that attributes effects solely to human side effects (e.g., biological, cognitive, social, and psychological ontologies) exposes soul care to power dynamics that supersede and devalue the cultural system.
- The principles of individual soul care and confession (e.g., with a sacramental underpinning, absolute secrecy, where the right to exercise the ministry as a priest stands and falls with absolute secrecy, not to be called to testify in court) makes research on effects unethical, in contrast to a randomized control–test methodology in psychiatry and psychology research.

Let us finally consider how military cultural socialization and adaptation on an identity perspective can impact priests who serve in Swedish military contexts.

## Cultural identity hybridization among military priests

Recent empirical interview research has illustrated that military priests can develop a kind of hybrid identity consisting of two contrasting identities: the priest and the military (Grimell, 2020b, 2021a, 2021b). Once again, it is important to emphasize the contextual dimension to understand this. In a Swedish context, the military priest is not only uniformed, he or she can, during deployment, choose to be combat equipped with a pistol and assault rifle for self-protection. Although this is optional, military culture tends to make the choice for them, as there is also a military identity socialization of the priests who prepare and participate during deployment (Grimell, 2021a).

### Military chaplains are socialized into a military culture

Military priests become part of military culture: not least symbolically through the uniform yet also physically, mentally, and emotionally. The cultural identity process that this entails does not escape priests, as everyone is affected (Grimell, 2020a, 2021a, 2022). This is especially the case because military culture is exclusive: not just anyone can become a member and is accepted in a particular group, platoon, company, battalion, or regiment or equivalent. It requires adaptation and developing specific military identities. A military priests’ identity is an example of a special kind of identity where the uniform symbolizes the military while the priests’ shirt symbolizes the church—a crossing of military and ecclesial identities (Grimell, 2020b, 2021a, 2021b). One aspect that reinforces the need for adaptation in such a process is that priests come from the outside. In Swedish research, military priests experienced themselves not only as guests in someone else’s house; they also represented another culture (Grimell, 2020b, 2021a). Many believed that they had to work purposefully, systematically, and in a committed way to build relationships, trust, and acceptance. Only then could they reach the soldier/sailor/officer behind the uniform and practice soul care in a way where the thresholds became low, and important existential conversations arose spontaneously in all possible situations (Grimell, 2020b, 2021a). This relationship-building process involves as military identity construction (the emergence of a military narrative about who I am as a military priest). When a priest has adapted to the degree that he or she felt accepted by the military group and organization, an identity as *military* also emerges (Grimell, 2021a; Grimell, 2021b).

The uniform symbolizes both a kind of adaptation to the military world of life and an identity claim for who I am in such a community and outside it. The wearing of the uniform can perhaps be considered a soft adaptation to a specific environment and simultaneously as the surface knowledge of the military context, which in itself is not particularly threatening. An adaptation involving a uniform and a priest’s shirt need not be too challenging either for a priest or other people outside the Swedish Armed Forces in the ecclesiastical context. But the closer one gets to the core of military culture, the greater the contrasts (Grimell, 2021a; Grimell, 2021b). For example, the church embraces brokenness and weakness, while military culture emphasizes power, strength, efficiency, endurance, and discipline. Military culture embraces extreme physical and mental performance, fitness, muscle, hard training, and combat training in order to be able to address combat tasks. The greater a cultural adaptation becomes, the more it is reflected in the identity claims that are made (Grimell, 2021a; Grimell, 2021b).

### Arms distinctly delineate the cultural boundary between ecclesiastical and military identities

A major adaptation step for military priests, and military identity development, is obviously weapons training, competency, or combatant training with weapons and armament during pre-deployment and during deployment to conflict zones, which also creates conditions for potentially killing in self-defense. Arms mark a clear cultural dividing line between ecclesiastical and military identities (Grimell, 2021a). The strategy or explanatory model that appeared in interviews to navigate critical discussions has been pragmatic—i.e., to carry weapons and assume the role of a soldier like everyone else during deployment in order to resolve their own self-protection. They used neither theological reasoning nor a theological legitimation. While this challenged their integrity as priests, it also created unique conditions for practicing soul care by being present (ministry of presence), truly understanding the military craft, and gaining trust and legitimacy from military personnel (Grimell, 2020a; Grimell, 2020b; Grimell, 2021a, 2021b). Thus, *military cultural competence* (Atuel and Castro, 2018) among deployed military priests is exceptional when conducting military soul care. It should be added that armed military priests only appear during deployments, not during military soul care carried out in Sweden—which sort of illustrates the normal situation and context of a military priest.

Integrity is challenged in a low-intensity way when a priest is in a military environment and is exposed to the values, meanings, and practices that prevail there. Such an influence slowly and stealthily builds up an identity as a military priest. Such an identity illustrates and represents elements of military culture: military words, expressions, values, meanings, and practices (Grimell, 2021a). The process is not quick, but in the long run, the results can be thorough. In order to protect the integrity during this process, a thorough selection process concerning military chaplains is required, a lived connection to the Church of Sweden, participation in an ecclesiastical context to practice soul care with a senior soul care giver, and education. All these elements are needed and function to safeguard that a priest can continue to be a priest in the Swedish Armed Forces, which may be easier said than done (Grimell, 2021a; Grimell, 2021b).

### Institutional commonalities

Although the emphasis is on cultural contrasts, there are also commonalities between these institutions, already seen and presented by the military sociologist Huntington (1957) in the 1950s in their analogy between a monastery and a military organization. The Church of Sweden and the Armed Forces are two of the oldest institutions in Sweden. They have lived side by side and collaborated since the nation was founded (Grimell, 2020a). Both organizations maintain traditions and commemorations that may coincide in a military–ecclesiastical practice and manifest as lived religion (Grimell, 2021a). Both institutions understand the value of tradition, ritual, drama, and liturgy, and can relate to this. Both institutions uphold something greater than the individual (Grimell, 2021a). Self-sacrifice and suffering are part of serving the greater: the collective, the unit, and the group. Giving one’s life for one’s neighbor is not unknown in the DNA of these institutions; in either the church (Bonhoeffer, 1959) nor in the armed forces (Franks, 2004; French, 2005; Huntington, 1957). A soldier’s integrity rests on the fact that he or she can be trusted to loyally address problems together with his battle buddies with his life at stake. Sacrificing one’s life for what one believes in, something larger than the self, is the foundation of a Christian tradition which has also shaped theology (Bonhoeffer, 1959). However, at their core, the institutions are bearers of contrasting cultures. Not least, ordination vows clearly indicate that a priestly identity is far from a military identity (Grimell, 2021a).

In contrast to the cultural context of the Church of Sweden, the requirements of war and combat form the core and ideological foundation of the Swedish Armed Forces. This means that the Swedish Armed Forces educates, trains and imposes accountability that military personnel must be able to comply with the requirements in terms of combat skills, techniques, and tactics, in all imaginable situations, in the performance of their duties. The armed forces foster military identities in line with the demands of war, the Geneva convention, and other rules of engagement, where an obvious part of military activity is to kill other people (combatants) if required. The cultural socialization is powerful and no human being, neither military nor priest, can escape being deeply affected by it (Grimell, 2021a, 2022). Education and training also contain strong rites of passage that mark changing identity events (before and after) and consolidate strong and robust military identities (French, 2005). Against the background of the construction of strong military identities and deployments to conflict or war zones, it can be said that the employer, the Swedish Armed Forces, has an influence over military personnel (Segal, 1986; Thornborrow and Brown, 2009; Verrips, 2006; Woodward and Jenkings, 2011). This influence extends to military priests as well. Moreover, this influence cannot be easily separated from life outside the military and the identities that are sheltered therein (Grimell, 2020b, 2021a, 2022).

## Final remarks

### The military society is not like the civilian one

The Swedish Armed Forces, like other armed forces, illustrates a particular cultural context in society with archaic and timeless contours that deal with killing one’s enemy (whoever that may be) (French, 2005; Goldstein, 2001; Strachan, 2006; Verrips, 2006; Wilson, 2008). However, in Sweden, unlike most other countries, the power dynamics between, for example, military medicine and psychiatry and military soul care are based on other historical, cultural, societal, political, military, and ecclesiastical conditions and considerations. This in turn has resulted in the power dynamics of military psychiatry taking on other embodied expressions in the Swedish Armed Forces. While military medicine and psychiatry are still characterized by the power position and normativity attributed to medicine in the rest of society, there is simultaneously a deep military understanding and need to address the multilayered stresses faced by military personnel during wartime which far exceed a purely military medical understanding of the human condition in a cultural and symbolic society. As part of such cultural complexity, military priests, through their military soul care, play a significant role in a Swedish military context in peace times, during crisis, and in war. The mismatch between the complexities and diversities of human experience and the certainty of scientific solutions, some of which are profitable, are considerably less pronounced in the Swedish Armed Forces than, for example, in the Swedish healthcare context, which embraces other perspectives and is dominated by the power dynamics of medical culture under which healthcare chaplains operate with a completely different and cropped mandates and purposes (Grimell and Bradby, 2022).

### It is crucial to be aware of the balancing act that arises in relation to medicine

The collaboration between medical personnel (within the practice of military medicine) and military chaplains in a Swedish context is not particularly well-developed, aside from occasional referrals to military chaplains (Grimell, 2021a). Mixed medical teams, however, are significantly more developed in a North American context; for example, in Canada (Smith-MacDonald et al., 2018). While there are benefits (Smith-MacDonald et al., 2018) there are also risks (Moss and Prince, 2014) to consider with mixed teams. Benefits can be attributed to a potentially lower risk of over-medicalizing social, psychological, spiritual, and existential problems for which medical methods are ineffective or have undesired side-effects (Illich, 1977). The risks can be attributed to the power dynamics which still operate in mixed healthcare teams. Such power dynamics can be reflected in, for example, a desire to adapt, classify, and understand matters that fall within an existential, spiritual, and religious realms in a psychiatric and psychological way and not on their own ontological terms (Moss and Prince, 2014). This also applies to the risk that a military chaplain can be seen as part of the medical team which also exercises *surveillance* (Foucault, 1979, 2006). The intermingling of medical personnel and military chaplains in one and the same team can impact the perception that they cooperate together (which they do) and undermine the absolute secrecy and integrity that a military priest has. The safe and absolutely protected relief valve provided by a military chaplain could lose both its integrity and importance. Also, it would be unethical for a priest to share information about, for example, the health issues of military personnel and in a Swedish context it would not be compatible with the Order of the Church (2024).

### Role distinctions

The Swedish model distinguishes between the roles of medical personnel and military chaplains. Medical personnel focus on addressing medical issues, while military chaplains provide for and address military soul care. Their responsibilities range from advising and supporting commanders on ethical and moral concerns, to training troops in ethics and morality. They also oversee war grave services and conduct ceremonies such as field sermons. In addition, they offer individual soul care and counseling with absolute confidentiality. Absolute secrecy functions to safeguard the integrity of military soul care. It protects it from the power dynamics of *surveillance* (Foucault, 1979), *medicalization* (Conrad, 2005; Conrad et al., 2010), and *commercial interests* (Bradby, 2012). This does not mean that dialog and discussion do not occur between clinicians and military priests; on the contrary, there are examples of such exchanges, but they involve only very generic information that does not in any way jeopardize the seal of absolute secrecy (Grimell, 2021a). They do embody different ontologies, epistemologies, and purposes while also respecting each other’s distinctiveness and contributions to the multilayered stresses in military activities, which far exceed a purely single-minded understanding of the human condition in a cultural and symbolic society. The deep military insight articulated in a Swedish context also indicates that a solid and strong value base, along with moral, ethical, existential, and spiritual support—provided through military soul care—is necessary to maintain one’s resilience during war (Swedish Armed Forces, 2024). This military perspective emphasizes the necessity of military priests practicing military soul care. It also acknowledges the benefits and deficits of medicine at both the individual and system levels. Additionally, it balances these aspects with other knowledge systems and moralities of care, support, and healing (Bradby, 2016).

### A culturally sensitive approach to military chaplaincy services

The final remark emphasizes the importance of a culturally sensitive approach to and understanding of military pastoral and spiritual care, as well as chaplaincy services worldwide. Military chaplains, along with their roles and practices, should not necessarily be viewed as a generic, cross-cultural phenomenon with many similarities or even shared concepts and expressions. Such an understanding is reductionist, as each country’s military spiritual care and chaplaincy services must be understood within its own historical and cultural context.

## Data Availability

The original contributions presented in the study are included in the article/supplementary material, further inquiries can be directed to the corresponding author.

## Footnotes

^1^A clear exception is war-torn Ukraine, which is currently in the process of building and professionalizing military chaplaincy services.

^2^There is very little historical research on Swedish military spiritual care currently available.

^3^Verified during a telephone with the Chief of Chaplains with the rank of Brigadier General, 8 March, 0900–1000. Notes were taken throughout the interview.

^4^This does not include all other religious groups, such as Catholic and Orthodox Christians, free churches and various faith movements, the relatively large Muslim population, Buddhists, Hindus, Jehovah’s Witnesses, and others.

^5^Verified during a telephone with the Chief of Chaplains with the rank of Brigadier General, 8 March, 0900– 1000. Notes were taken throughout the interview.

^6^The duties within the framework of military soul care presented by the Chief of Chaplains at a conference for Swedish military priests at the Swedish Armed Forces’ Head Quarters, March 12, at 1500–1600.

## References

[ref1] Antonovsky A. (1987). Unraveling the mystery of health: How people manage stress and stay well. San Francisco, CA: Jossey-Bass.

[ref2] Atuel H. R. Barr N. Jones E. Greenberg N. Williamson V. Vermetten E.. (2021). Understanding moral injury from a character domain perspective. J. Theor. Philos. Psychol. 41, 155–173. doi: 10.1037/teo0000161

[ref3] Atuel H. R. Castro C. A. (2018). Military cultural competence. Clin. Soc. Work. J. 46, 74–82. doi: 10.1007/s10615-018-0651-z

[ref4] Bobrow J. Cook E. Knowles C. Vieten C. (2013). Coming all the way home: integrative community care for those who serve. Psychol. Serv. 10, 137–144. doi: 10.1037/a0031279, PMID: 23244031

[ref5] Boisen A. T. (1936). The exploration of the inner world. New York, NY: Harper and Brothers.

[ref6] Bonhoeffer D. (1959). The cost of discipleship. New York, NY: MacMillan.

[ref7] Bradby H. (2012). Medicine, health and society: A critical sociology. London: SAGE.

[ref8] Bradby H. (2016). Research agenda in medical sociology. Front. Sociol. 1:14. doi: 10.3389/fsoc.2016.00014

[ref9] Braidotti R. (2006). Transpositions. Cambridge: Polity Press.

[ref10] Bryan C. J. Graham E. Roberge E. (2015). Living a life worth living: spirituality and suicide risk in military personnel. Spirituality Clin. Prac. 2, 74–78. doi: 10.1037/scp0000056

[ref11] Cabot R. C. Dicks R. L. (1936). The art of ministering to the sick. New York, NY: Macmillan.

[ref12] Capps D. (2008). Jesus the village psychiatrist. Louisville, KY: Westminister John Knox Press.

[ref13] Capps D. Fowler G. (2010). The pastoral care case. Eugene, ON: Wipf & Stock.

[ref14] Church of Sweden in Statistics. (2024). Available at: https://www.svenskakyrkan.se/statistik (Accessed April 4, 2024).

[ref15] Conrad P. (2005). The shifting engines of medicalization. J. Health Soc. Behav. 46, 3–14. doi: 10.1177/002214650504600102 15869117

[ref16] Conrad P. Mackie T. Mehrotra A. (2010). Estimating the costs of medicalization. Soc. Sci. Med. 70, 1943–1947. doi: 10.1016/j.socscimed.2010.02.019 20362382

[ref17] Courts of Sweden. (2024). Available at: https://www.domstol.se/amnen/kallad-till-domstol/Att-vittna/ (Accessed April 4, 2024).

[ref18] Danbolt L. J. (2019). Chaplaincy: special issue. Nordic J. Prac. Theol. 36, 1–3.

[ref19] Dicks R. L. (1944). Pastoral work and personal counseling. New York, NY: Macmillan.

[ref20] Foucault M. (1979). Discipline and punish: The birth of prison. New York, NY: Vintage books.

[ref21] Foucault M. (2006). Psychiatric power: Lectures at the Collège de France, 1975–1976. London: Picador.

[ref22] Franks T. (2004). American soldier: General Tommy Franks. New York, NY: Regan Books.

[ref23] French S. (2005). The code of the warrior: Exploring warrior values past and present. Lanham, MD: Rowman & Littlefield Publishers.

[ref24] Ganzevoort R. R. (2009). Forks in the road when tracing the sacred: Practical theology as hermeneutics of lived religion. Paper presented at the International Academy of Practical Theology, Chicago. Available at: https://www.researchgate.net/publication/238070309_Forks_in_the_Road_when_Tracing_the_Sacred_Practical_Theology_as_Hermeneutics_of_Lived_Religion (Accessed August 3, 2009).

[ref25] Ganzevoort R. R. Roeland J. H. (2014). Lived religion. The praxis of practical theology. Int. J. Pract. Theol. 18, 91–101. doi: 10.1515/ijpt-2014-0007

[ref26] Goldstein J. (2001). War and gender: How gender shapes the war system and vice versa. Cambridge: Cambridge University Press.

[ref9001] Gore J. M. (1995). On the continuity of power relations in pedagogy. Int. Stud. Sociol. Educ. 5, 165–188. doi: 10.1080/0962021950050203

[ref27] Government Offices in Sweden. (2024). Available at: https://www.regeringen.se/regeringskansliet/regeringskansliets-anstallda/ (Accessed April 4, 2024).

[ref28] Graham L. K. (2017). Moral injury: Restoring wounded souls. Nashville: Abingdon Press.

[ref29] Grimell J. (2020a). Military chaplaincy in Sweden: a contemporary perspective. J. Health Care Chaplain. 28, 81–94. doi: 10.1080/08854726.2020.1745490, PMID: 32233976

[ref30] Grimell J. (2020b). An interview study of experiences from pastors providing military spiritual care within the Swedish armed forces. J. Health Care Chaplain. 28, 162–178. doi: 10.1080/08854726.2020.1796077, PMID: 32715945

[ref31] Grimell J. (2021a). Uniformer, identiteter och militära själavårdare: En intervjustudie om prästers erfarenheter av institutionssjälavård i Försvarsmakten. Skellefteå: Artos Academic.

[ref32] Grimell J. (2021b). I-position as a tool to advance the understanding of pastors and deacons who navigate contrasting identities as chaplains: a narrative analysis. J. Health Care Chaplain. 28, 310–327. doi: 10.1080/08854726.2021.1886741 33645450

[ref33] Grimell J. (2022). The invisible wounded warriors in a nation at peace: An interview study on the lives of Swedish veterans of foreign conflicts and their experiences with PTSD, moral injuries, and military identities. Zürich: Lit Verlag.

[ref34] Grimell J. (2023a). Veteranhälsans limbo: En intervjustudie om ett försämrat mående och ökat lidande hos svenska utlandsveteraner. Skellefteå: Artos Academic.

[ref35] Grimell J. (2023b). Evil, constructed: a salient part of an emerging spiritual veteran identity. J. Pastoral Care Counseling 77, 148–157. doi: 10.1177/15423050231213418, PMID: 37946528 PMC10704872

[ref36] Grimell J. Bradby H. (2022). The dynamics of spiritual care among Swedish hospital chaplains: approaching the future in the present. Health Soc. Care Chaplaincy 10, 9–26. doi: 10.1558/hscc.18737

[ref37] Gudmundsson D. (2014). Konfessionell krigsmakt: Predikan och b€on i den svenska armen 1611-1721 (doktorsavhandling, Lunds universitet, Lund, Sverige). [A confessional armed force: Sermon and prayer within the Swedish Army 1611–1721] (Doctoral dissertation). Malmö: Universus Academic Press.

[ref38] Hansson K. (2016). Kyrkan i fält: Fältpräster i det svenska försvaret (organisation, principer och konflikter i ekumenisk belysning sedan 1900) [the church under arms: Military chaplains within the Swedish armed forces - organization, principles and conflicts with ecumenical aspects since 1900]. Skellefteå: Artos.

[ref39] Huntington S. (1957). The soldier and the state: The theory and politics of civil-military relations. Cambridge, MA: The Belknap Press of Harvard University Press.

[ref9002] Illich I. (1977). Limits to Medicine: Medical Nemesis: The Expropriation of Health. Harmondsworth: Penguin. 10.1136/jech.57.12.928 PMC1732345 14652253

[ref40] Kasselstrand I. (2015). Nonbelievers in the church: a study of cultural religion in Sweden. Sociol. Relig. 76, 275–294. doi: 10.1093/socrel/srv026

[ref41] Kilshaw S. (2011). Impotent warriors: Gulf war syndrome, vulnerability and masculinity. New York, NY: Berghahn Books. 10.1080/13648470903356836 27269915

[ref42] Koenig H. G. Carey L. B. Wortham J. S. (2023). Moral injury: A handbook for military chaplains. Manchester: Amazon Books.

[ref43] Kurkiala M. (2019). När själen går i exil: modernitet, teknologi och det heliga. Stockholm: Verbum AB.

[ref44] Kyrkans Tidning. (2021). Available at: https://www.kyrkanstidning.se/nyhet/valdagen-har-kommer-valresultaten (Accessed April 4, 2024).

[ref45] Larsson L. O. (2002). Gustav Vasa – landsfader eller tyrann? Stockholm: Prisma.

[ref46] Lee L. J. (2018). Moral injury reconciliation: A practitioner’s guide for treating moral injury, PTSD, grief, and military sexual trauma through spiritual formation strategies. London: Jessica Kingsley Publishers.

[ref47] Lemos C. Puga-Gonzalez I. (2021). Belief in god, confidence in the church and secularization in Scandinavia. Secular. Nonreligion 10, 1–21. doi: 10.5334/snr.143

[ref48] Litz B. T. Stein N. Delaney E. Lebowitz L. Nash W. P. Silva C.. (2009). Moral injury and moral repair in war veterans: a preliminary model and intervention strategy. Clin. Psychol. Rev. 29, 695–706. doi: 10.1016/j.cpr.2009.07.003, PMID: 19683376

[ref49] Liuski T. Grimell J. (2022). The professional competence of the Finnish and Swedish military chaplains in divergent operational environments: an international comparison. Relig. Worldviews Educ. 2, 74–96.

[ref50] McGuire M. (2008). Lived religion: Faith and practice in everyday life. New York, NY: Oxford, University Press.

[ref51] McLaren M. (2002). Feminism, Foucault, and embodied subjectivity. Albany: State University of New York (SUNY) Press.

[ref52] Moss P. Prince M. J. (2014). Weary warriors: Power, knowledge, and the invisible wounds of soldiers. New York, NY: Berghahn Books.

[ref53] National Church Office. (2024). Available at: https://www.svenskakyrkan.se/kyrkokansliet-i-uppsala (Accessed April 4, 2024).

[ref54] Order of the Church. (2024). Available at: https://www.svenskakyrkan.se/kyrkoordningen (Accessed April 4, 2024).

[ref55] Pargament K. I. (2008). The sacred character of community life. Am. J. Community Psychol. 41, 22–34. doi: 10.1007/s10464-007-9150-z 18080744

[ref56] Pargament K. I. (2011). Spiritually integrated psychotherapy: Understanding and addressing the sacred. New York, NY: The Guilford Press.

[ref57] Pargament K. I. Sweeney P. J. (2011). Building spiritual fitness in the Army: an innovative approach to a vital aspect of human development. Am. Psychol. 66, 58–64. doi: 10.1037/a0021657, PMID: 21219049

[ref58] Parsons T. (1991). The social system, Routledge sociology classics. 2nd Edn. London: Routledge.

[ref59] Ringmar R. (2002). Gustaf Eriksson Vasa: kung, kamrer, koncernchef: Återblick på en monark av Guds nåde. Stockholm: Atlantis.

[ref60] Saarelainen S. M. Peltomäki I. Vähäkangas A. (2019). Healthcare chaplaincy in Finland. Nordic J. Prac. Theol. 36, 22–45. doi: 10.48626/tpt.v36i2.5350

[ref61] Segal M. W. (1986). The military and the family as greedy institutions. Armed Forces Soc. 13, 9–38. doi: 10.1177/0095327X8601300101

[ref62] Shay J. (2002). Odysseus in America: Combat trauma and the trails of homecoming. New York, NY: Scribner.

[ref63] Smith-MacDonald L. A. Morin J.-S. Brémault-Phillips S. (2018). Spiritual dimensions of moral injury: contributions of mental health chaplains in the Canadian Armed Forces. Front. Psych. 9:592. doi: 10.3389/fpsyt.2018.00592, PMID: 30487762 PMC6246733

[ref64] Stallinga B. A. (2013). What spills blood wounds spirit: chaplains, spiritual care, and operational stress injury. Ref. Prac. Form. Supervision Ministry 33, 13–31.

[ref65] Strachan H. (2006). Morale and modern war. J. Contemp. Hist. 41:217. doi: 10.1177/0022009406062054

[ref66] Swedish Agency for Support to Faith Communities. (2024). Available at: https://www.myndighetensst.se/andlig-vard/andlig-vard-i-sjukvarden-idag.html (Accessed April 4, 2024).

[ref67] Swedish Armed Forces. (2024). Available at: https://www.forsvarsmakten.se/sv/var-verksamhet/militar-sjalavard/ (Accessed April 4, 2024).

[ref68] Thomsen K. Hvidt N. C. Søndergaard J. (2019). New wine in new leather bags? Nordic J. Prac. Theol. 36, 46–59. doi: 10.48626/tpt.v36i2.5352

[ref69] Thornborrow T. Brown A. D. (2009). Being regimented: aspiration, discipline and identity work in the British parachute regiment. Organ. Stud. 30, 355–376. doi: 10.1177/0170840608101140

[ref70] Tick E. (2005). War and the soul: Healing our nation’s veterans from post-traumatic stress disorder. Wheaton, IL: Theosophical Publishing House.

[ref71] Verrips J. (2006). “Dehumanization as a double-edged sword” in Grammars of identity/alterity: A structural approach. eds. Baumann G. Gingrich A. (New York, NY: Berghahn Books), 145–150.

[ref72] Wilson P. (2008). Defining military culture. J. Mil. Hist. 72, 21–22. doi: 10.1353/jmh.2008.0041

[ref73] Woodward R. Jenkings N. (2011). Military identities in the situated accounts of British military personnel. Sociology 45, 252–268. doi: 10.1177/0038038510394016

[ref74] World Value Survey Cultural Map. (2024). Available at: https://www.worldvaluessurvey.org/WVSNewsShow.jsp?ID=467 (Accessed April 4, 2024).

[ref75] Wortmann J. H. Eisen E. Hundert C. Jordan A. H. Smith M. W. Nash W. P.. (2017). Spiritual features of war-related moral injury: a primer for clinicians. Spirituality Clin. Prac. 4, 249–261. doi: 10.1037/scp0000140

